# A commentary on ‘Survival benefit of liver resection following complete response to transarterial chemoembolization for intermediate-stage hepatocellular carcinoma: a retrospective, multicenter, cohort study’

**DOI:** 10.1097/JS9.0000000000001223

**Published:** 2024-02-21

**Authors:** Chunhua Rui, Longhuan Piao, Peng Wang

**Affiliations:** a Institute of Molecular Medicine (IMM), Renji Hospital, Shanghai Jiao Tong University School of Medicine, Shanghai Jiao Tong University; b Department of Oncology, Fudan University Shanghai Cancer Center, Shanghai Medical College, Fudan University; c School of Life Sciences, Fudan University, Shanghai; d Department of Hepatobiliary and Pancreatic Surgery, Harbin Medical University Cancer Hospital, Harbin, Heilongjiang, People’s Republic of China

*Dear Editor,*

It was with great interest that we read the article published in the *International Journal of Surgery*. Hu *et al.* ^1^ conducted a multicenter retrospective cohort analysis of intermediate-stage hepatocellular carcinoma (HCC) patients with complete response to transcatheter arterial chemoembolization (TACE) followed by persistent observation (TACE group) or liver resection (LR) (TLR group). The results of the trial showed that intermediate-stage HCC patients could benefit from LR following the complete response to TACE, resulting in longer overall survival (OS) and disease-free survival (DFS).

HCC is a heterogeneous disease with multiple etiologies, accounting for 70–85% of primary liver cancers^2,3^. As the most prevalent liver cancer, it is also the sixth most common cancer and the third leading etiology of cancer-related deaths worldwide. HCC has a poor prognosis, with advanced-stage patients having a median survival of 6–8 months and terminal-stage patients having a median survival of less than 3–4 months, according to the updated Barcelona Clinic Liver Cancer (BCLC) staging system. Recurrence rates in resected HCC are still over 10% after a year and between 70 and 80% after 5 years. Treatment options range from radical treatments like radiofrequency ablation (RFA), transplantation, resection, and combination therapy to palliative treatments like TACE, chemotherapy, molecular target therapy, immunotherapy with immune checkpoint inhibitors, and supportive care, depending on the patient’s liver function and the stage of the cancer^4^.

TACE is commonly utilized for patients with intermediate-stage HCC, according to the Asia-Pacific Primary Liver Cancer Expert (APPLE) and Japan Society of Hepatology (JSH) consensus statements^5^. Conventional TACE (cTACE) has demonstrated a survival advantage in certain HCC patients, but the technique has significant drawbacks, including a very high incidence of systemic toxicity and varying procedure standards. TACE-resistant tumors, such as confluent multinodular, poorly differentiated, or diffuse type HCCs, may also benefit from selective TACE once systemic drugs have been administered. Drug-eluting bead TACE (DEB-TACE), which employs sophisticated technology to provide a more controlled release and prolonged concentration of chemotherapeutics in patients, has grown in popularity as a TACE therapy for unresectable HCC.

This study sought to assess the survival benefit of LR after a full response to TACE for intermediate-stage HCC. To summarize, intermediate-stage HCC patients may benefit from LR after a complete response to TACE, resulting in extended OS and DFS. Furthermore, patients with scores higher than 7 may not benefit from LR therapies. Several factors should be considered: this was a retrospective study with a small sample size, which may have resulted in some bias in the results; this is still an exploratory study. The therapy should be verified with a longer follow-up period and a multicenter prospective trial.

## Ethical approval

This manuscript is a comment. Don’t need ethical approval.

## Consent

This manuscript is a comment. Don’t need patient consent.

## Author contribution

C.R. and L.P.: study concept or design, data collection, data analysis or interpretation, and writing the paper; P.W.: study concept or design, writing, and revising the paper.

## Conflicts of interest disclosure

This manuscript is a comment without conflicts of interest.

## Research registration unique identifying number (UIN)

This manuscript is a comment. Don’t need UIN.

## Guarantor

Peng Wang.

## Data availability statement

This manuscript is a comment. Don’t need a Data availability statement. However, all the data from the current study are publicly available.

## Provenance and peer review

This manuscript is a comment without being invited.
